# Mortality Rates Across 25-Hydroxyvitamin D (25[OH]D) Levels among Adults with and without Estimated Glomerular Filtration Rate <60 ml/min/1.73 m^2^: The Third National Health and Nutrition Examination Survey

**DOI:** 10.1371/journal.pone.0047458

**Published:** 2012-10-24

**Authors:** Holly Kramer, Chris Sempos, Guichan Cao, Amy Luke, David Shoham, Richard Cooper, Ramon Durazo-Arvizu

**Affiliations:** 1 Department of Preventive Medicine and Epidemiology, Loyola University Medical Center, Maywood, Illinois, United States of America; 2 Department of Medicine, Loyola University Medical Center, Maywood, Illinois, United States of America; 3 Division of Nephrology and Hypertension, Loyola University Medical Center, Maywood, Illinois, United States of America; 4 Office of Dietary Supplements, National Institutes of Health, Bethesda, Maryland, United States of America; Innsbruck Medical University, Austria

## Abstract

**Background:**

Previous studies exploring the association between 25[OH]D levels and mortality in adults with and without kidney disease utilized 25[OH]D thresholds that have recently been scrutinized by the Institute of Medicine Committee to Review Dietary References Intakes for Vitamin D and Calcium.

**Objective:**

We explored all-cause mortality rates across the spectrum of 25[OH]D levels over an eighteen-year follow-up among adults with and without an estimated glomerular filtration rate (eGFR) <60 ml/min/1.73 m^2^.

**Design:**

The study included 1,097 U.S. adults with eGFR <60 ml/min/1.73 m^2^ and 14, 002 adults with eGFR ≥60 ml/min/1.73 m^2^. Mortality rates and rate ratios (RR) across 25[OH]D groups were calculated with Poisson regression and restricted cubic splines while adjusting for covariates.

**Results:**

Prevalence of 25[OH]D levels <30 and <20 ng/ml among adults with eGFR <60 ml/min/1.73 m^2^ was 76.5% (population estimate 6.2 million) and 35.4% (population estimate 2.9 million), respectively. Among adults with eGFR ≥60 ml/min/1.73 m^2^, 70.5% had 25[OH]D levels <30 ng/ml (population estimate 132.2 million) while 30.3% had 25[OH]D levels <20 ng/ml (population estimate 56.8 million). Significantly higher mortality rates were noted among individuals with 25[OH]D levels <12 ng/ml compared to referent group (24 to <30 ng/ml): RR1.41 (95% CI 1.17, 1.71) among individuals with eGFR <60 ml/min/1.73 m^2^ and RR 1.32 (95% CI 1.13, 1.56) among individuals with eGFR ≥60 ml/min/1.73 m^2^ after adjustment for covariates including co-morbid conditions. Mortality rates were fairly similar across all 25[OH]D groups with levels >20 ng/ml after adjustment for all covariates.

**Conclusions:**

Regardless of presence of eGFR <60 ml/min/1.73 m^2^, mortality rates across groups with 25[OH]D levels 20–40 ng/ml are similar.

## Introduction

25-Hydroxyvitamin D (25[OH]D) deficiency is based on 25[OH]D levels associated with rickets in children [1] although there currently is no consensus on thresholds that define 25[OH]D deficiency or insufficiency. For example, the Institute of Medicine Committee to Review Dietary References Intakes for Vitamin D and Calcium defines “risk of deficiency” as levels <12 ng/ml [2] and that some, but not all, individuals may be at “risk for insufficiency” with 25[OH]D levels between 12 to 20 ng/ml. The Endocrine Society Clinical Practice Guideline defines 25[OH] deficiency and insufficiency as levels <20 ng/ml and levels 20 to 29 ng/ml, respectively. [3] The existing controversy over 25[OH]D levels which define “insufficiency” or “risk of insufficiency” requiring supplementation carries substantial public health relevance because approximately half of the U.S. non-institutionalized adult population has a 25[OH]D level within the range of 12–29.9 ng/ml.l [4], [5] Thus, the majority of 25[OH]D supplementation is currently for treatment of “insufficient” rather than “deficient” levels of 25[OH]D.

**Figure 1:**
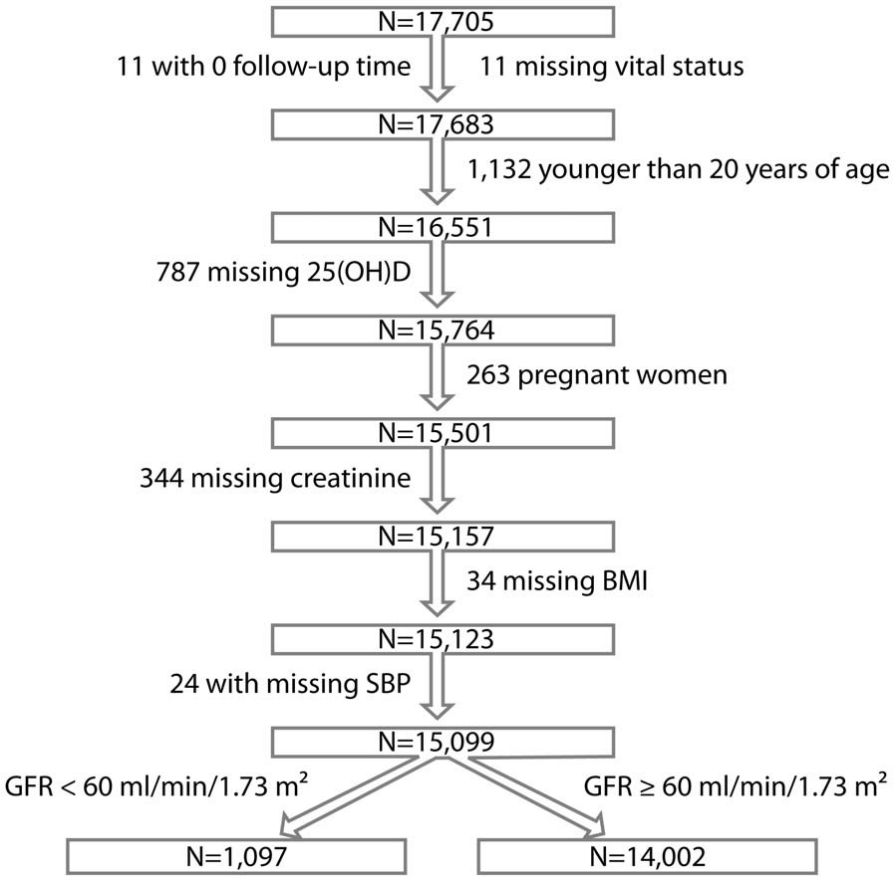
Flowchart of individuals included in the analysis of the association between 25[OH]D and all-cause mortality.

Two previously published studies examined the association between 25[OH]D levels and mortality in adults with CK [4], [5] and without CKD [5] using the Third National Health and Nutrition Examination Study linked with the mortality file. These studies utilized either 25[OH]D quartiles or categories of 25[OH]D based on previous treatment thresholds that define 25[OH]D deficiency as <15 ng/ml, and 25[OH]D insufficiency as levels 15−<30 ng/ml. These 25[OH]D groups were then compared to a group with 25[OH]D levels >30 ng/ml. This 25[OH]D cut-point (>30 ng/ml) is based on 25[OH]D levels whereby parathyroid hormone (PTH) levels are maximally suppressed in adults without CKD. [4]–[9] This treatment threshold has been questioned by the Institute of Medicine Committee to Review Dietary References Intakes for Vitamin D and Calcium, a committee convened by the Canadian and U.S. governments. The Committee concluded that 25[OH]D levels ≥20 ng/ml are sufficient for the majority of individuals. [2] Observational studies have shown heightened risk for both mortality and cardiovascular outcomes with 25[OH]D levels <20 ng/ml, but 25[OH]D levels are strongly influenced by sun exposure, skin pigmentation, and body fat. These factors may co-vary with co-morbid conditions that impact overall mortality. Given the recent reappraisal of clinical thresholds for 25[OH]D supplementation, [2] we examined all-cause mortality rates across the range of 25[OH]D levels in adults with and without eGFR <60 ml/min/1.73 m^2^, which defines stage 3–5 CKD, using a representative sample of U.S. adults. The population was stratified by presence of stage 3–5 CKD due to the strong link between disorders of mineral metabolism, co-morbid conditions which influence vitamin D levels (through decreased outdoor activity and alterations in diet) and reduced glomerular filtration rate. [10] Models were constructed to determine how baseline co-morbid conditions may influence the association between baseline 25[OH]D levels and all-cause mortality.

## Materials and Methods

### Study Population

The study population source was the Third National Health and Nutrition Examination Survey (NHANES III) linked with the National Death Index with up to 18 years of follow-up (1988–2006). The NHANES III was designed as a probability sample of the total civilian non-institutionalized population, 2 months of age or older, in the United States and collected health and nutritional data on 33,994 men, women, and children from 1988–1994. Certain subgroups were over-sampled such as young children, older persons, non-Hispanic blacks, and Mexican Americans. The survey was approved by the National Center for Health Statistics Institutional Review Board and all participants provided written informed consent. Details of the survey design may be found in the NHANES III operations manual. [11] All NHANES III participants who were 17 years and older at the time of the survey were eligible for mortality follow-up. This study was approved by the Loyola University Medical Center Institutional Review Board.

Figure 1 outlines the selection of NHANES participants included in the final analysis. A total of 17, 683 adults age 20 years and older with known vital status were available for analysis and 864, 344 and 263 were excluded for missing information on 25[OH]D levels, serum creatinine and pregnancy status, respectively. An additional 58 individuals were excluded due to missing information on covariates. The analysis was then limited to 1,097 participants with eGFR <60 ml/min/1.73 m^2^ (defined below) and 14,002 with eGFR ≥60 ml/min/1.73 m^2^.

**Table 1:** Baseline characteristics of study participants with eGFR<60 ml/min/1.73 m^2^ by 25[OH]D groups.

	25-Hydroxyvitamin D Groups (ng/mL)						
	<12 (n = 102)	12–16(n = 146)	17–19(n = 187)	20–24(n = 198)	25–29(n = 230)	30–39(n = 183)	40+ (n = 51)
**25[OH]D (ng/mL)**	9.9(0.24)	14.3(0.94)	17.9(0.11)	22.0(0.12)	26.9(0.15)	34.4(0.27)	45.4(1.4)
**Age (years)**	70.6(3.5)	74.1(1.4)	74.6(0.8)	72.9(1.4)	72.4(1.1)	72.9(1.3)	69.4(4.1)
**% Male**	25.1(7.8)	23.8(4.7)	31.9(4.1)	39.4(5.7)	45.0(4.1)	54.2(4.7)	55.3(10.7)
**Race/Ethnicity**							
** % NH-White**	71.2(6.3)	76.2(4.6)	87.9(2.0)	89.0(2.7)	91.7(1.7)	85.2(4.9)	90.4(5.9)
** % NH-Black**	21.8(4.8)	18.3(3.9)	8.4(1.6)	5.6(1.1)	5.6(1.0)	4.3(1.1)	3.2(1.8)
** % Mexican**	4.1(1.3)	1.2(0.4)	2.1(0.6)	0.7(0.3)	0.8(0.3)	0.6(0.3)	0.5(0.3)
** % Other Race**	2.8(2.1)	4.3(2.3)	1.6(0.9)	4.7(2.3)	1.9(1.4)	9.9(5.0)	5.9(5.7)
**% Diabetes**	25.0(6.4)	25.3(4.4)	14(2.8)	17(3.6)	16(2.7)	10(1.5)	6(3.7)
**% Previous MI**	17.1(4.4)	20.1(3.9)	17.4(4.0)	14.4(3.9)	18.9(3.9)	16.5(3.6)	6.3(3.4)
**% Previous CHF**	15.4(4.9)	13.6(3.4)	15.0(3.4)	3.31(3.2)	14.6(3.5)	11.9(3.0)	4.0(2.5)
**% Previous Stroke**	17.0(5.6)	2.3(3.1)	9.4(3.2)	6.8(2.1)	12.1(2.8)	11.4(2.9)	3.1(1.8)
**% Cancer**	7.9(4.0)	8.0(2.6)	11.4(3.3)	12.8(3.2)	8.7(2.1)	13.9(2.7)	5.1(3.2)
**% Current Smokers**	15.1(3.1)	12.6(4.4)	9.5(3.0)	16.4(4.5)	8.2(3.1)	12.5(4.3)	11.5(5.5)
**BMI(kg/m^2^)**	28.4(0.8)	27.7(0.7)	28.0(0.6)	28.1(0.7)	27.1(0.4)	26.5(0.6)	24.4(0.5)
**SBP(mmHg)**	143.4(3.0)	145.5(2.2)	143.6(2.0)	144.1(1.8)	142.4(2.0)	142.9(1.8)	141.9(2.5)
**eGFR(mL/min/1.73 m^2^)**	46.6(1.6)	44.8(1.2)	47.2(1.2)	49.7(0.6)	49.1(0.8)	49.5(0.6)	49.1(2.2)
**Calcium (mg/dL)**	9.3 (0.07)	9.4 (0.07)	9.3 (0.04)	9.3 (0.05)	9.3 (0.05)	9.4 (0.05)	9.4 (0.09)
**Phosphorous (mg/dL)**	3.7 (0.08)	3.6 (0.06)	3.5 (0.04)	3.5 (0.04)	3.5 (0.06)	3.4 (0.04)	3.4 (0.11)
**Education**							
** % Less HS**	53.1(9.6)	45.1(5.6)	45.2(6.2)	52.2(5.3)	44.4(4.0)	54.9(4.6)	41.8(9.6)
** % Completed HS**	20.6(4.2)	32.3(5.6)	32.4(4.9)	26.7(4.1)	24.7(4.2)	25.7(3.4)	18.6(7.6)
** % More HS**	26.3(8.3)	22.4(4.8)	22.3(4.8)	21.0(4.0)	30.9(4.2)	19.3(3.3)	39.6(10.4)

* Data are shown as mean (standard error) or percentages (standard error).

### Estimated Glomerular Filtration Rate

Serum creatinine was measured by a Roche/Hitachi 737 analyzer (Roche Diagnostics, Indianapolis, IN using the kinetic alkaline picrate reaction. [12] Serum creatinine values calibrated to standardized creatinine values [13] were used to estimate glomerular filtration rate (eGFR) with the Chronic Kidney Disease-Epidemiology Collaboration (CKD-EPI) estimating equation:

eGFR = 141* min(Scr/κ, 1)^α^ * max(Scr/κ, 1)^−1^.^209^ *0.993^Age^ *1.018 [if female]*1.159 [if African American] [14].where κ is 0.7 for females and 0.9 for males, α is −0.329 for females and −0.411 for males, min indicates the minimum of Scr/κ or 1, and max indicates the maximum of Scr/κ or 1.

### Mortality

Mortality data was obtained from the NHANES III Linked Mortality File Public-use file. [15] This file contains mortality status through December 31, 2006 for participants 17 years of age and older who participated in the NHANES III survey. Mortality status was ascertained from the National Death Index or Social Security Administration records. In some cases, death of NHANES III participants was also verified by reviewing death certificates and comparing them with survey records to confirm the matching of a death record of a survey participant with the National Death Index record. Deaths occurring during years 1988–1998 were coded according to the International Classification of Diseases, 9^th^ revision, Clinical Modification. [16] All other deaths were coded according to the International Classification of Diseases, 10^th^ Revision. [17] Cause of death was grouped according to ICD-10.

**Table 2:** Baseline characteristics of adults with eGFR ≥60 ml/min/1.73 m^2^ by 25(OH)D groups.

	25-Hydroxyvitamin D Groups (ng/mL)						
Characteristic	<12 (n = 1419)	12–16(n = 2194)	17–19(n = 2603)	20–24(n = 2328)	25–29(n = 2816)	30–39(n = 1973)	40+ (n = 669)
**25(OH)D (ng/mL)**	9.8(0.08)	14.1(0.04)	18.0(0.04)	22.1(0.03)	26.9(0.04)	34.2(0.08)	46.9(0.48)
**Age (years)**	44.0(0.7)	45.3(0.7)	45.6(0.6)	45.1(0.7)	44.2(0.5)	41.6(0.6)	38.6(0.8)
**% Male**	31.0(1.6)	35.8(1.9)	43.6(1.8)	48.6(1.4)	52.3(1.4)	58.0(1.2)	54.4(2.4)
**Race/Ethnicity**							
** % NH-White**	35.8(2.8)	51.8(2.8)	64.6(2.0)	77.7(1.6)	83.2(1.2)	91.1(0.9)	95.3(0.9)
** % NH-Black**	46.4(2.5)	27.1(1.7)	15.6(1.1)	8.0(0.7)	4.3(0.4)	2.2(0.3)	0.9(0.2)
** % Mexican**	7.1 (0.7)	7.8(0.8)	7.7(0.8)	6.1(0.6)	4.8(0.5)	3.0(0.4)	1.4(0.3)
** % Other Race**	10.8(2.7)	13.3(1.7)	12.1(1.4)	8.3(1.1)	7.8(1.0)	3.7(0.8)	2.3(0.9)
**% Winter**	59.7(4.4)	52.8(4.9)	47.7(4.4)	38.3(4.6)	38.4(4.4)	34.7(4.4)	27.6(5.2)
**% Diabetes**	4.5(0.6)	9.4(0.9)	6.0(0.8)	5.7(0.8)	4.7(0.5)	2.6(0.4)	2.3(0.9)
**% Previous MI**	1.6(0.4)	3.5(0.6)	3.1(0.6)	3.7(0.5)	2.9(0.4)	2.1(0.4)	2.0(0.6)
**% Previous CHF**	1.2(0.3)	2.4(0.5)	1.5(0.4)	2.1(0.4)	1.7(0.3)	1.2(0.2)	0.7(0.3)
**% Previous Stroke**	2.3(0.6)	2.4(0.7)	1.9(0.4)	1.5(0.3)	1.3(0.2)	1.1(0.3)	1.5(0.6)
**% Cancer**	3.0(0.5)	2.9(0.7)	4.3(0.6)	3.7(0.7)	3.5(0.4)	3.2(0.5)	3.4(0.9)
**% Current Smoking**	36.7(2.5)	32.1(1.7)	28.4(1.5)	27.6(1.7)	26.1(1.3)	29.2(1.9)	34.8(2.4)
**BMI(kg/m^2^)**	28.0(0.4)	28.1(0.2)	27.7(0.3)	26.9(0.2)	26.3(0.2)	25.4(0.2)	24.5(0.2)
**SBP(mmHg)**	123.6(0.9)	124.0(0.6)	123.8(0.5)	122.2(0.6)	121.5(0.5)	119.8(0.7)	118.6(0.9)
**eGFR(mL/min/1.73 m^2^)**	107.6 (0.8)	103.2(0.9)	100.3(0.7)	100.4(0.7)	100.0(0.5)	100.3(0.7)	101.6(0.7)
**Calcium (mg/dL)**	9.2 (0.03)	9.2 (0.02)	9.2 (0.02)	9.3 (0.02)	9.3 (0.03)	9.3 (0.03)	9.3 (0.03)
**Phosphorous (mg/dL)**	3.5(0.02)	3.5(0.02)	3.5(0.02)	3.4 (0.02)	3.4 (0.02)	3.4 (0.01)	3.4 (0.02)
**Education**							
** % Less HS**	26.8(1.3)	26.9(1.8)	26.0(1.6)	24.4(1.3)	23.8(1.3)	19.7(1.9)	19.8(2.5)
** % Completed HS**	38.9(2.3)	37.3(1.7)	32.8(1.6)	33.4(1.2)	31.3(1.1)	34.7(1.5)	33.8(2.2)
** % More HS**	34.3(2.4)	35.8(1.9)	41.2(2.0)	42.2(1.5)	44.9(1.6)	45.6(2.5)	46.4(3.3)

### Vitamin D Levels

In NHANES III, higher latitude regions (northern states) were sampled during the summer, whereas lower latitude regions (southern states) were generally sampled during the winter. Serum 25[OH]D measurements were completed at the National Center for Environmental Health, Centers for Disease Control using a radioimmunoassay kit (DiaSorin, Stillwater, MN) [18]. The inter-assay coefficient of variation (CV) differed across 25[OH]D levels with the highest CV noted among those with 25[OH}D values <25 ng/ml (15–25%) and the lowest CV noted among those with 25[OH]D values >80 mmol/L (14–18%). Seven groups of 25[OH]D levels (ng/ml) were created with the lowest group defined as 25[OH]D <12 ng/ml consistent with 25[OH]D deficiency**.** [2] Levels of 25[OH]D levels above this threshold were then divided into 6 additional groups: 12–15.9, 16–19.9, 20.0–23.9, 24–29.9, 30.0–39.9, ≥40 ng/ml.

**Figure 2:**
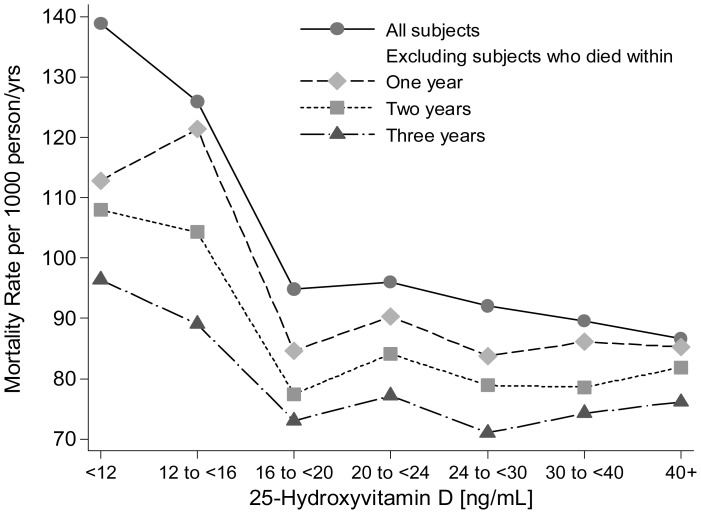
Age-, sex-, season- and race/ethnicity-adjusted mortality rates per 1,000 person-years by 25(OH)D groups among adults with eGFR <60 ml/min/1.73 m^2^. Figure 2 includes all participants (with eGFR <60 ml/min/1.73 m^2^) and under 3 exclusion strategies: 1) Exclusion of participants who died within one year of baseline examination; 2) Exclusion of participants who died within two years of baseline examination; 3) Exclusion of participants who died within three years of baseline examination. Rates were computed using Poisson regression analysis after grouping 25[OH] D values into 7 groups.

**Figure 3:**
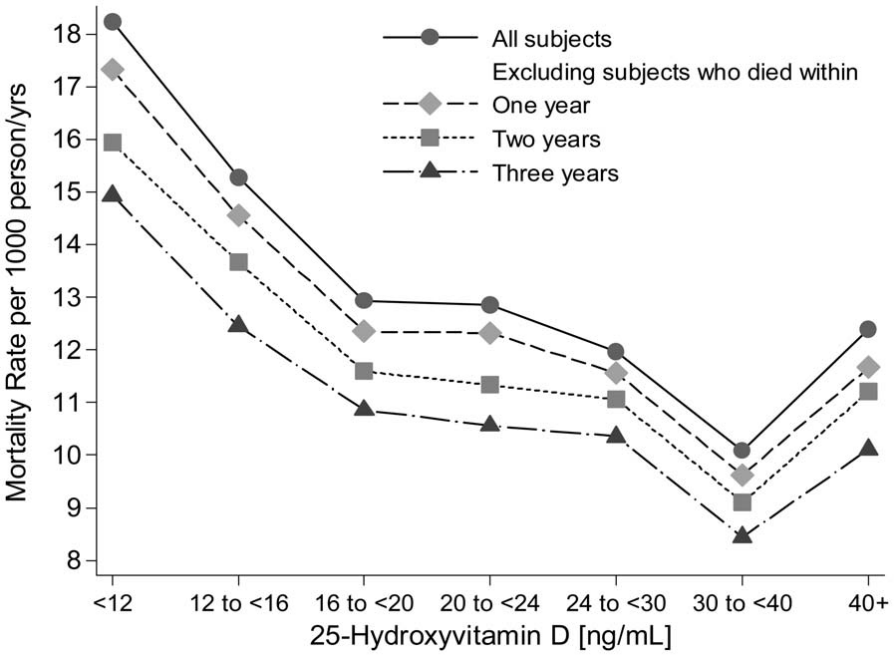
Age-, sex-, season- and race/ethnicity-adjusted mortality rates per 1,000 person-years by 25(OH)D groups among adults without eGFR <60 ml/min/1.73 m^2^. Figure 3 includes all participants (without eGFR <60 ml/min/1.73 m^2^) and under 3 exclusion strategies: 1) Exclusion of participants who died within one year of baseline examination; 2) Exclusion of participants who died within two years of baseline examination; 3) Exclusion of participants who died within three years of baseline examination. Rates were computed using Poisson regression analysis after grouping 25[OH] D values into 7 groups.

### Covariates

Age was defined as the age at the time of the interview, and race/ethnicity was self-reported as non-Hispanic white, non-Hispanic black, or Mexican-American. Other race/ethnicities were grouped into “Other”. Trained examiners measured blood pressure using standardized protocols and the average of all six blood pressure measurements collected during the interview and physical exam was used for this analysis. Diabetes was defined as self-reported previous physician diagnosis other than during pregnancy or the current or past use of glucose lowering agents. Presence of congestive heart failure, non-skin related cancer, stroke, and myocardial infarction were based on self-report. Participants provided a single spot urine specimen and urine albumin and creatinine were measured using a solid-phase fluorescence immunoassay and the Jaffé rate reaction (Beckman Astra, Brea, CA), respectively. Urine albumin/creatinine ratios in mg/g were calculated for all participants. Serum phosphate was measured using a Hitachi model 737 multi-channel analyzer (Boehringer Mannheim Diagnostics, Indianapolis, IN). Use of anti-hypertensive medications or anticonvulsants, estrogens, or glucocorticoids was based on self-report. The season in which the examination took place was dichotomized as winter (October-March) or summer (April-September). Education was defined as less than high school, high school completion or education past high school. Cigarette smoking was defined as current smoking (yes/no).

**Table 3:** Total number of deaths by eGFR status 25[OH]D groups.

		25[OH]D Groups (ng/mL) GFR<60 ml/min/1.73 m^2^						
Cause of Death	Total	<12 (n = 102)	12–16 (n = 146)	17–19 (n = 187)	20–24 (n = 198)	25–29 (n = 230)	30–39 (n = 183)	40+ (n = 51)
**All Causes**	902	88	125	149	168	182	148	42
**Cancer**	117	10	14	16	22	31	19	5
**Major CVD**	487	52	63	82	91	96	79	24
**Ischemic HD**	285	32	34	46	55	53	50	15
**Stroke**	82	6	14	10	17	20	14	1
**Other CVD**	120	14	15	26	19	23	15	8
**Infections Disease**	111	9	18	23	17	18	21	5
**Diabetes**	30	2	6	4	8	5	5	0
**GI**	5	0	1	0	3	0	1	0
**Renal**	33	3	8	5	8	7	2	0
**Accidents**	19	2	3	3	3	3	4	1
**Other/Unknown**	279	24	45	48	52	52	46	12
		**25[OH]D Groups (ng/mL) GFR≥60 ml/min/1.73 m^2^**						
	**<12 (n = 1419)**	**12–16 (n = 2194)**	**17–19 (n = 2603)**	**20–24 (n = 2328)**	**25–29 (n = 2816)**	**30–39 (n = 1973)**	**40+ (n = 669)**	
**All Causes**	2882	288	467	545	500	593	385	104
**Cancer**	709	57	115	135	128	141	100	33
**Major CVD**	1173	112	211	229	187	242	159	33
**Ischemic HD**	285	32	34	46	55	53	50	15
**Stroke**	82	6	14	10	17	20	14	1
**Other CVD**	120	14	15	26	19	23	15	8
**Infections Disease**	347	35	52	56	68	66	51	19
**Diabetes**	96	17	15	23	11	20	10	0
**GI**	56	10	7	17	8	10	2	2
**Renal**	20	5	4	1	5	4	1	0
**Accidents**	126	15	16	21	24	28	16	6
**Other/Unknown**	355	37	48	65	69	81	46	11

### Statistical Analysis

NHANES III data are weighted to account for the probability of selection and to adjust for non-response to the interview and physical exam. All the analyses were conducted using STATA 12 (STATA Corporation, College Station, TX) “Survey” procedures which incorporate the weights, strata and cluster of the complex study design. Descriptive characteristics of adults with and without eGFR <60 ml/min/1.73 m^2^ were reported across the seven groups of 25[OH]D levels. Person-months of follow-up were calculated from the date of the survey interview through the date of death, or to the end of the mortality follow-up period, December 31, 2006. Adjusted mortality rates per 1000-person years were calculated for each 25[OH]D category using Poisson regression and restricted cubic splines. For each 25[OH]D group the median 25[OH]D level of the interval and the subject specific values of the covariates were placed into the estimated regression equation. The mortality rate is the average rate over all subjects in the 25[OH]D group. Mortality rate ratios were calculated for each 25[OH]D group compared to the referent 25[OH]D group (24–29.9 ng/mL).

We estimated the mortality rate ratio for six 25[OH]D groups compared to the reference 25[OH]D group (24–29.9 ng/ml). This group was selected as the referent because it includes 25[OH]D levels which are above the threshold for “risk of insufficiency” defined by the Institute of Medicine Committee to Review Dietary References Intakes for Vitamin D and Calcium [2] yet below the thresholds defined as “insufficient” in previous analyses. [4], [5] This group also includes values consistent with average 25[OH]D levels in adult men and women. The median 25[OH]D value was estimated for each of these 6 groups (10.0 14.1, 18.0, 21.9, 26.5, 33.9, and 43.6 ng/ml), with the median for the 24–29.9 group as the reference (26.5 ng/ml). Thus, the mortality rate ratio of an individual with 25[OH]D = 14.1 ng/ml compared to the reference value, 26.5 ng/ml, represents the mortality rate ratio of all subjects in the group<12–15.9 ng/ml compared to the group 24–29.9 ng/ml. Similar calculations were carried out for the other groups.

**Table 4:** All-cause mortality rate per 1000 Person-Years (95% CI) and mortality rate ratios by 25[OH]D groups among participants with eGFR<60 ml/min/1.73 m^2^.

	25[OH]D Groups (ng/mL)						
	<12	12 to <16	16 to <20	20 to <24	24 to <30	30 to <40	40+
**Median 25[OH]D value**	**(10.0)**	**(14.1)**	**(18.0)**	**(21.9)**	**(26.5)**	**(33.5)**	**(43.6)**
* **Rate per 1000 PY (95% CI)**	153 (126, 180)	121 (105, 138)	108 (93, 123)	108 (95, 121)	108 (93, 123)	100 (88, 111)	97 (78, 115)
**Rate Ratio (95% CI)**	1.41 (1.17, 1.71)	1.12 (0.91, 1.38)	1.00 (0.82, 1.22)	1.00 (0.91, 1.09)	Ref	0.92 (0.79, 1.08)	0.89 (0.72, 1.12)

* All-cause mortality rate per 1000 person-years.

Model adjusts for age, sex, race/ethnicity, season of 25[OH]D measurement, co-morbidities, BMI, SBP, eGFR, smoking, medication use and educational level.

**Table 5:** All-cause mortality rate per 1000 person-years (95% CI) and mortality rate ratios by 25[OH]D groups among participants with e GFR≥60 ml/min/1.73 m^2^.

	25[OH]D Groups (ng/mL)						
	<12	12 to <16	16 to <20	20 to <24	24 to <30	30 to <40	40+
**Median 25[OH]D value**	**(10.0)**	**(14.1)**	**(18.0)**	**(21.9)**	**(26.5)**	**(33.5)**	**(43.6)**
* **Rate (95% CI)**	17 (15, 19)	14 (13, 16)	13 (12, 15)	13 (12, 14)	13 (11, 14)	11 (10, 13)	12 (10, 15)
**Rate Ratio (95% CI)**	1.32 (1.13, 1.56)	1.123 (0.95, 1.33)	1.04 (0.89, 1.21)	1.03 (0.96, 1.12)	Ref	0.89 (0.77, 1.02)	0.96 (0.79, 1.17)

* All-cause mortality rate per 1000 person-years.

Model adjusts for age, sex, race/ethnicity, season of 25[OH]D measurement, co-morbidities (cancer, MI, CHF, stroke, diabetes), BMI, SBP, eGFR, smoking, medication use and educational level.

After testing the assumptions of the proportional hazards model by using log minus log survival plots, analyses were repeated using Cox-proportional hazard models and compared with results using Poisson regression. The model adjusted for for age. sex, race,season, co-morbidities (diabetes, CHF, MI, stroke, cancer) body mass index (BMI), systolic blood pressure (SBP), eGFR, smoking status, use of anti-hypertensive medications, anticonvulsant medications, estrogens or glucocorticoids, spot urine albumin/creatinine ratios, serum phosphorous and education. The addition of spot urine albumin/creatinine ratios did not change the parameter estimate for the association between 25[OH]D group and all-cause mortality. Because spot urine albumin/creatinine ratios were missing in a substantial number of participants and did not confound the association between 25[OH]D groups and all-cause mortality after adjusting for all other covariates, this variable was not included in the final model in order to maximize the total number of individuals for the analyses. Age, BMI, SBP, serum phosphorous and eGFR were placed in the model as continuous variables while all other covariates were fitted as categorical variables. To further explore potential confounding by co-morbid conditions, analyses with 25[OH]D as categories and as a continuous variable were repeated after excluding deaths which occurred within one, two or three years after the follow-up visit. All p values were two sided with values <0.05 considered statistically significant. The number of deaths for NHANES groups with and without eGFR<60 ml/min/1.73 m^2^ provided adequate precision for the analyses given the suggested rule of 10 outcomes per covariate in the model. [19].

**Figure 4:**
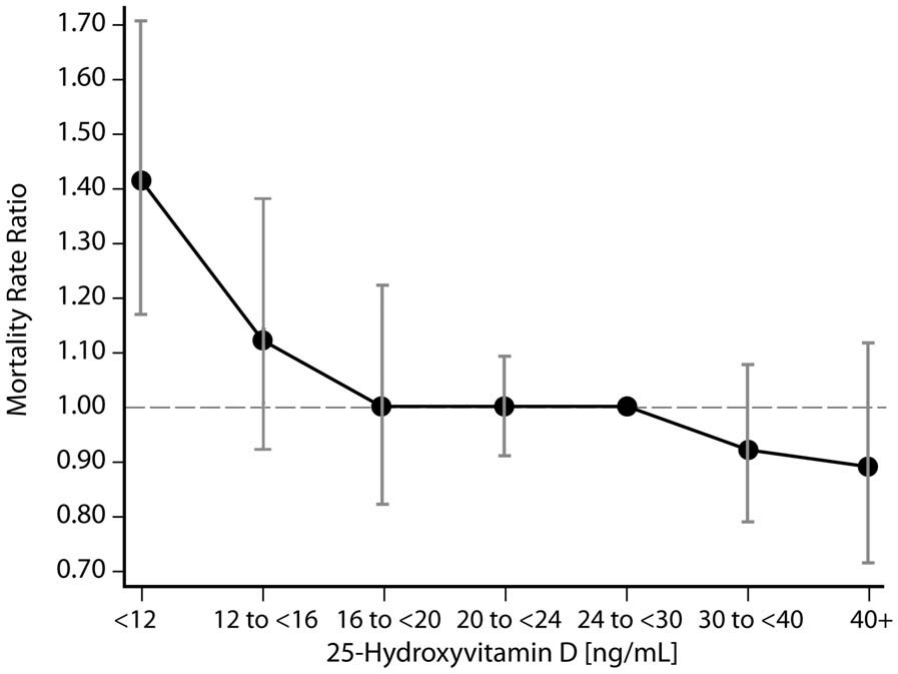
Adjusted mortality rate ratios by 25[OH]D groups for participants with eGFR<60 ml/min/1.73 m^2^. Rate ratios were computed using Poisson regression analysis with 25[OH]D modeled via restricted cubic splines, and covariates age, sex, season, race/ethnicity, co-morbidities (history of diabetes, congestive heart failure, stroke, and myocardial infarction), education status, smoking, systolic blood pressure, eGFR, body mass index. The median of the fifth 25[OH]D group (24 ng/mL−29.9 ng/mL) was used as the referent value. Error bars represent 95% confidence intervals.

**Figure 5:**
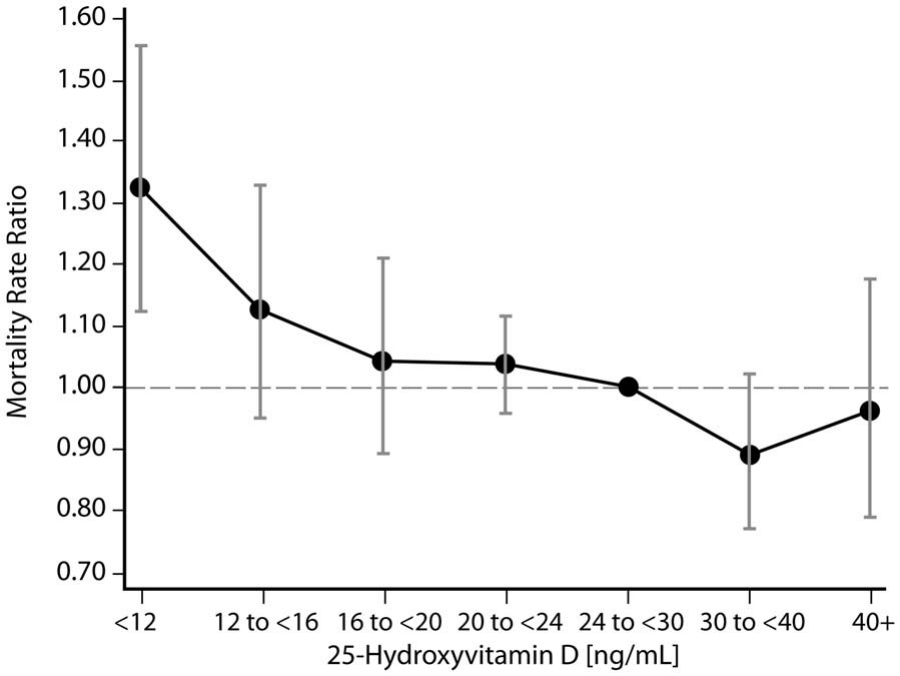
Adjusted mortality rate ratios by 25[OH]D groups for participants with eGFR≥ ml/min/1.73 m^2^. Rate ratios were computed using Poisson regression analysis with 25[OH]D modeled via restricted cubic splines, and covariates age, sex, season, race/ethnicity, co-morbidities (history of diabetes, congestive heart failure, stroke, and myocardial infarction), education status, smoking, systolic blood pressure, eGFR, body mass index. The median of the fifth 25[OH]D group (24 ng/mL−29.9 ng/mL) was used as the referent value. Error bars represent 95% confidence intervals.

## Results

The study population consisted of 15,099 adults (1,097 with eGFR <60 ml/min/1.73 m^2^ and 14,002 with eGFR ≥60 ml/min/1.73 m^2^). Among the individuals with eGFR <60 ml/min/1.73 m^2^, 1,011 had an eGFR 59–30, 58 had eGFR 29–15, and 28 had an eGFR <15 ml/min/1.73 m^2^. Participants with eGFR <60 ml/min/1.73 m^2^ were older and more likely to female and have co-morbid conditions (previous myocardial infarction, congestive heart failure, stroke and cancer). The median 25[OH]D value in adults with and without eGFR <60 ml/min/1.73 m^2^ was 22.3 ng/ml (interquartile range 16.5–28.9) and 21.3 ng/ml (interquartile range 15.8–28.0), respectively. Among adults with eGFR ≥60 ml/min/1.73 m^2^, 70.5% had 25[OH]D levels <30 ng/ml (population estimate 132.2 million) while 30.3% had 25[OH]D levels <20 ng/ml (population estimate 56.8 million). Prevalence of 25[OH]D levels <30 and <20 ng/ml among adults with eGFR <60 ml/min/1.73 m^2^ was 76.5% (population estimate 6.2 million) and 35.4% (population estimate 2.9 million), respectively.

Tables 1 and 2 show participant characteristics by 25[OH]D groups among those with and without eGFR <60 ml/min/1.73 m^2^, respectively. Among adults with eGFR <60 ml/min/1.73 m^2^, those in the lowest 25[OH]D group (<12 ng/ml) had the lowest average eGFR and were older, and more likely to be female and non-white compared to participants in the 25[OH]D groups ≥12 ng/ml. Those in the lowest 25[OH]D group also had higher average BMI, SBP, phosphorus level, and higher prevalence of diabetes, hypertension, and prior myocardial infarction compared to the other 25[OH]D groups. Prevalence of current smoking and BMI were higher among participants with eGFR ≥60 ml/min/1.73 m^2^ in the lowest 25[OH]D group compared to the groups with 25[OH]D levels >17 ng/ml while prevalence of co-morbid conditions did not differ substantially across 25[OH]D groups in this population.

Table 3 shows the number of deaths and reported cause of death by 25[OH]D groups among adults with and without eGFR <60 ml/min/1.73 m^2^. The majority of deaths for adults with and without eGFR <60 ml/min/1.73 m^2^ were attributed to cardiovascular diseases while cancer was the second leading reported cause. Figures 2 and 3 show the mortality rates per 1,000 person-years by 25(OH)D groups for adults with and without eGFR <60 ml/min/1.73 m^2^, respectively, after adjustment for age, sex, race/ethnicity and season of 25[OH]D measurement. Figures includes all participants (with and without eGFR <60 ml/min/1.73 m^2^ for Figures 2 and 3, respectively) and after excluding participants who died during the first year of follow-up, the first two years of follow-up, or the first three years of follow-up. After excluding these early deaths, the overall mortality rate within each 25[OH]D group decreased with the exclusion of deaths during the first one, two or three years of follow-up. However, the overall distribution of deaths across 25[OH]D groups did not change substantially.

Tables 4 and 5 show all-cause mortality rates per 1000 person-years and mortality rate ratios by 25[OH]D groups. Among adults with eGFR <60 ml/min/1.73 m^2^, the fully-adjusted all-cause mortality rates ranged from 153 per 1000 person-years (95% CI 126 to 180 per 1000 person-years) in the 25[OH]D group<12 ng/ml to 121 per 1000 person-years (95% CI 105 to 138 per 1000 person-years) in the next highest 25[OH]D group (12− <16 ng/ml). Fully adjusted mortality rates were then very similar across groups with 25[OH]D levels >20 ng/ml. Lowest mortality rates were noted in the highest 25[OH]D group at 97 deaths per 1000 person-years (95% CI 78 to 115 per 1000 person-years).

Among adults with eGFR ≥60 ml/min/1.73 m^2^, fully adjusted mortality rates per 1000 person-years were over seven-fold lower compared to adults with eGFR <60 ml/min/1.73 m^2^ for all 25[OH]D groups. Fully adjusted mortality rates ranged from as high as 17 per 1000 person-years (95% CI 15 to 19 per 1000 person-years) in the lowest 25[OH]D group (<12 ng/ml) to as low as 11 per 1000 person-years (95% CI 10 to 13 per 1000 person-years) in the group with 25[OH]D levels 30 to <40 ng/ml. Fully adjusted mortality rates were very similar across 25[OH]D groups with levels between 20 to >40 ng/ml.

Figures 4 and 5 demonstrate rates of all-cause mortality across 25[OH]D groups relative to the referent 25[OH]D group (24 to <30 ng/ml) among adults with and without eGFR <60 ml/min/1.73 m^2^, respectively. For all adults, significant differences in fully adjusted mortality rates were only noted for the group with 25[OH]D levels <12 ng/ml compared to the referent 25[OH]D group (24 to <30 ng/ml). Mortality rates were 41% higher among individuals with 25[OH]D levels <12 ng/ml and eGFR <60 ml/min/1.73 m^2^ (95% CI 1.17, 1.71) and 32% higher among individuals with 25[OH]D levels <12 ng/ml and eGFR ≥60 ml/min/1.73 m^2^ (95% CI 1.13, 1.56). However, no significant differences in mortality rates relative to the referent group were noted for any of the other 25[OH]D groups after adjustment for all covariates among all adults. Similar results were also noted when hazard ratios for mortality were calculated by 25[OH]D groups using the Cox proportional hazards model (data not shown).

## Discussion

In a representative sample of the US adults with and without eGFR <60 ml/min/1.73 m^2^, 25[OH]D levels <12 ng/ml, consistent with 25[OH]D deficiency, [2] are associated with significantly increased risk of all-cause mortality after adjustment for all covariates. These findings are consistent with previous studies which examined the association between 25[OH]D levels and mortality using data from the Third National Health and Nutrition Examination Survey. [4], [5] The persistence of the association between 25[OH]D deficiency and mortality after adjustment for co-morbid conditions and the consistent shape of the overall distribution of all-cause mortality across 25[OH]D groups after eliminating participants who died during the first three years of follow-up argues against 25[OH]D deficiency simply reflecting overall poor health. These findings are supported by several observational studies which show that 25[OH]D deficiency is associated with an increased risk of mortality in adults with and without CKD.[4], [5], [20]–[23] A meta-analysis of observational studies showed that the association between 25[OH]D and mortality appears non-linear with no significant decreases in mortality once 25[OH]D levels exceed 35 ng/ml compared to levels <11 ng/ml. [23] In the meta-analysis which included 62, 548 individuals from 14 prospective studies, baseline 25[OH]D levels were <20 ng/ml in the majority of these studies. [23] Most, [4], [5], [23], [24] but not all studies [25] have demonstrated that low 25[OH]D levels are associated with heightened mortality risk. However, it is difficult to compare results across studies due to differing cut-points for defining low or high 25[OH]D levels. In addition, most previous studies compared low 25[OH]D levels to high 25[OH]D levels. In this study, the referent group contained the average 25[OH]D level among U.S. adults. The majority of adults with and without CKD in industrialized societies are not 25[OH]D deficient but rather have levels of 25[OH]D between 12–30 ng/ml. [10], [20], [26], [27] Indeed, vitamin D supplementation for 25[OH]D levels between 20–30 ng/ml will require treating approximately 3.3 and 75.4 million additional adults with and without eGFR <60 ml/min/1.73 m^2^, respectively, compared to supplementation for 25[OH]D levels <20 ng/ml. Differences in mortality rates across the range of 25[OH]D levels between 20 to 40 ng/ml were quite small but clinical trials are needed to determine the benefits and risks of 25[OH]D supplementation.

In the last several years, numerous studies have demonstrated an association between low levels of 25[OH]D and chronic diseases.[1], [22], [28]–[32] Evidence for 25[OH]D as a causal factor for most of these diseases remains inconclusive. Data from clinical trials suggest that 25[OH]D supplementation with ergocalciferol (vitamin D [2]) or cholecalciferol (vitamin D [3]) is associated with a relative risk reduction of 7%. [33] However, the association between 25[OH]D supplementation and mortality differs by baseline serum 25[OH]D levels, which is not consistent across trials. [33] Thus, optimal levels for treatment goals remain undetermined. Moreover, 25[OH]D supplementation may increase the risk of cancers [34], [35] and kidney stones. [36] Given the results of previous supplementation trials of other vitamins or minerals, [37]–[39] higher levels may not necessarily be beneficial and may heighten risk despite suggestion of clinical utility from observational studies. Clinical trials are needed to ascertain the risks and benefits of vitamin D supplementation for adults with and without kidney disease.

The strengths of this study include the representative sample of non-institutionalized U.S. adults with known survey weights, allowing us to extrapolate to the general population. However, these findings are not generalizable to adults living in nursing homes or those receiving dialysis or who have received a kidney transplant. Measurement of 25[OH]D occurred during the warmer months in the Northern states, which may potentially underestimate the prevalence of 25[OH]D deficiency and insufficiency in this region. Optimal thresholds where mortality rate is lowest could not be determined due to low numbers of individuals in this group. Although 25(OH)D exists in two forms [25(OH)D2 and 25(OH)D3], the Diasorin assay measures only total 25(OH)D. [40] Finally, because our study is observational, we cannot rule out confounding by unmeasured factors such as social deprivation. [41].

In conclusion, while significantly higher mortality rates are noted with 25[OH]D levels <12 ng/dl, mortality rates are fairly similar across the range of 25[OH]D levels 20–40 ng/dl among adults with and without eGFR <60 ml/min/1.73 m^2^.
